# Inequality of public facilities between urban and rural areas and its driving factors in ten cities of China

**DOI:** 10.1038/s41598-022-17569-2

**Published:** 2022-08-02

**Authors:** Ronghua Xu, Wenze Yue, Feiyang Wei, Guofu Yang, Yi Chen, Kaixuan Pan

**Affiliations:** 1 Department of Land Management, Zhejiang University, Zijingang Campus, 866 Yuhangtang Road, Hangzhou, 310058 Zhejiang People’s Republic of China; 2 School of Design and Environment, National University of Singapore, Singapore, 117566 Singapore; 3 Artistic Design & Creation School, Zhejiang University City College, Hangzhou, 310015 People’s Republic of China; 4 College of Life Sciences, Zhejiang University, Hangzhou, 310058 People’s Republic of China; 5 Institute of Environmental Science, Leiden University, Einsteinweg 2, 2333 CC Leiden, The Netherlands

**Keywords:** Socioeconomic scenarios, Sustainability

## Abstract

Urban development continues to face the dilemma of spatial inequality of public facilities, particularly educational and medical facilities. Identifying inequalities in various types of public facilities and their driving mechanisms is crucial in reducing social inequality. However, information on this topic is limited. This study took 10 typical cities in China as cases. We used the methods of the Gini coefficient and hedonic price model as bases in evaluating the equality of nine types of education and medical facilities, focusing on the differences between urban and rural areas. Moreover, we further analyzed the driving factors of facility equality. Results showed that equality of public facilities in urban areas was significantly higher than that in rural areas. Primary schools, middle schools, and health service centers were relatively equal, and kindergartens and pharmacies were unequal only in rural areas. However, the equality of facilities with large-size or commercial attributes was not optimistic. Furthermore, there remained a significant gap among counties (or districts), which was mainly driven by population, economy, and building density in the form of logarithm and logarithmic linear models. Our research contributes to an in-depth understanding of the inequality of public facilities and further supports decision-making to improve social equality.

Urban facilities provide various services locally and even across regions, making a positive contribution to the quality of life of urban residents^1–3^. However, there remain the serious problem of spatial differentiation in the supply of urban public facilities^4–6^, such as the evident urban–rural duality, particularly in developing or middle-income countries, thereby resulting in social injustice^7^. In the past two decades, numerous studies have considerably focused on the spatial distribution, accessibility, and equality of urban green and gray facilities^8–10^, especially the research on the benefits of urban green space has been relatively mature^11–13^. However, grey facilities closely related to socioeconomic activities are equally important, providing residents with a series of services such as residence, transportation, education, medical treatment, entertainment, and public security^1,6,14,15^. Moreover, social equality of education and medical facilities is imminent under the circumstances of the current adjustment of population fertility driven by global population aging and the COVID-19 pandemic^16,17^. In recent years, China has implemented the development strategy of public service equalization and new urbanization^18,19^, highlighting the key issues of coordinated development of public facilities in urban and rural areas^19^. Previous studies have made important contributions to the equality assessment of specific geographical regions or for some types of public facilities^1,20,21^, such as the comprehensive evaluation framework based on accessibility^2,22^. However, there remains a lack of systematic analysis on the equality differences and driving mechanisms between urban and rural regions, even on multiple spatial scales^13,21^. Although the imbalance of public facilities may constantly exist, reducing the inequality is related to the improvement of residents’ well-being and regional coordinated development.

Theoretically, spatial equality originates from social equality and is generally defined as human expectations for the distribution of service facilities^23,24^. That is, residents must be treated equally no matter where they live in the city^24,25^. Previous studies have shown that urban facilities are rarely distributed evenly in the entire urban space, which is also applicable to the population pattern^26^. In fact, the distribution of most urban facilities on the city center-edge gradients shows a power function or logarithmic decline^26^. The current urban structure mostly expands with some emerging modes, such as polycentric urban patterns and urban sprinkling structure^27,28^. Even so, high density information and material flow continue to gather in the central area of a city, connected to the surrounding decentralized structure through urban facilities^3,29^. Given the massive migration of rural population to urban areas, built-up areas continue to expand to the suburbs^30^, resulting in an aggravation of the living gap between urban and rural residents^15,27,31^. When dividing the urban–rural boundary, the majority of related studies have adopted the density grade of impervious layer, the boundary of night lights, the concentration area of population density or administrative boundary of a city to explore the facility pattern, service distribution^2,11^, and landscape pattern on the urban–rural gradient^2,30,32,33^. Nevertheless, we lack substantial knowledge of the cross-comparison of public facility inequality in different geographical regions, particularly between urban and rural areas, which is not conducive to the reasonable allocation of public facilities.

At present, the research on the spatial equality of urban facilities has mainly focused on the distribution characteristics^10,34^, accessibility^35^, and inequality of single or a few types of facilities in specific areas^5,25^. In general, all urban facilities are important to residents’ daily life, but their importance varies^36,37^. Some facilities are needed every day^38^ (e.g., primary schools), while others are enjoyed on weekends or holidays^12^ (e.g., public parks). The spatial equality of education and health facilities is not only an issue of immense concern to urban researchers and planners but also residents^1,6^. Interestingly, urban residents have different demand preferences for supporting facilities at different levels or sizes^19^, either focusing on distance factors (e.g., clinics and pharmacies) or preferring service quality^39,40^ (e.g., hospitals). However, in the current research on the equality of education or medical facilities, more people tend to quantitatively evaluate the spatial differences of inequality of a certain type of facilities^15,22^, often disregarding the fact that even facilities with similar functions have differences between different levels or types of facilities^4,19^. In addition, urban sustainability is multidimensional and multi-scale in nature, and solutions focusing on one spatial scale or dimension may not produce the same effect on different spatial scales or dimensions^20,41^. Although the research on the spatial equality of education and medical facilities has involved numerous cities or regions in China, such as Shanghai^42^, Hangzhou^25^, Wuhan^40^, Fuzhou^23^, and Eastern China^43,44^, the revealed equality remains limited to the identification of local facility inequality.

Indicators to quantify the spatial inequality of facilities generally include the Gini coefficient^2^, location entropy^19^, and accessibility and its matching degree with social economy^5,45^ (e.g., residents’ income and house price). Numerous previous studies have measured the inequality of facility distribution by measuring the accessibility index of facilities^12,46^ (i.e., distance index). However, accessibility distance based on facilities could not objectively reflect the matching degree between population density and resource distribution^7,20^. Note that the Gini coefficient was originally used in economics to measure and analyze the income inequality^7^, and then gradually applied to measure the supply equality of various public services^40,47^, and the key is that it can be used to compare temporal and spatial differences. Previous studies have indicated that in addition to demographic factors, the spatial equality of public facilities is closely related to various socioeconomic factors^26,44^. Some studies have attempted to analyze the correlation between spatial inequality of public facilities and socioeconomic variables, such as population density, population characteristics, economic income, land price, and other variables representing regional differences^19,48^. Unfortunately, understanding how facility equality changes complex with spatial or socioeconomic gradients remains relatively limited^4,19^. Moreover, as a method to deal with the relationship between various characteristics and prices of heterogeneous products, hedonic price model (HPM) is widely used in real estate and commodity markets to quantify the relationship among spatial variables^49,50^. Therefore, a worthy endeavor is to attempt to gain inspiration from HPM to explore the response relationship and quantitative ways of spatial equality of facilities and resources to multiple socioeconomic factors.

This study took 10 typical cities in China as cases and attempted to explore the spatial equality and their driving mechanisms of various educational and medical facilities in urban and rural areas. The three objectives are as follows: (1) assess the equality of nine types of education and medical facilities in case cities; (2) reveal the inequality differences of various facilities between urban and rural areas, counties, and regions; (3) determine the main driving factors affecting the change of spatial equality of various facilities, and quantify the relationship between equality and driving factors by establishing HPM. This study aims to deepen human understanding of the current situation of social equality and its development mechanism, and further provide empirical support for government managers and urban planners to reduce social inequality in urban and rural areas, and promote the sustainable and coordinated development of urban areas from multiple spatial scales.

## Results

### Equality of various public facilities in the entire city

**Figure 1:**
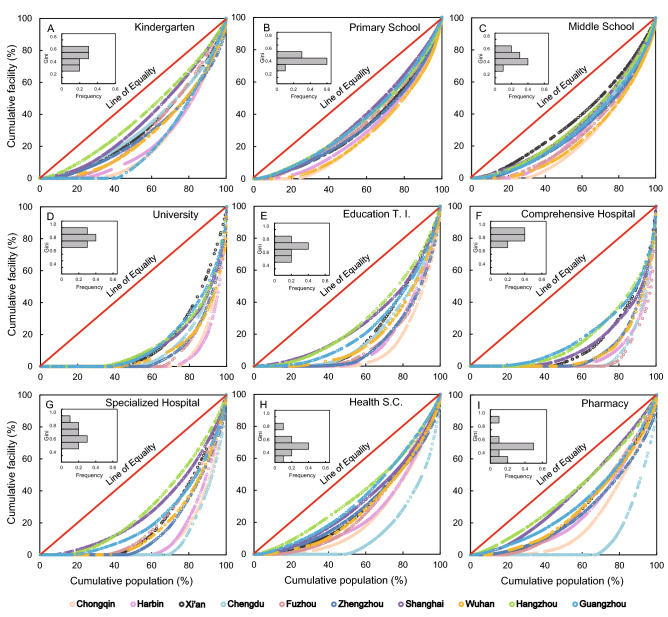
Lorenz curves of medical and educational facilities of 10 cities in China. Horizontal axis: cumulative percentage of population. Vertical axis: percentage of various educational and medical facilities corresponding to the proportion of population. Education T. I. in E represents the abbreviations of education-training institutions, and the Health S. C. in H represents the abbreviations of health service centers. Each color represents a city. The red line on the diagonal represents the Lorentz curve in the case of complete fairness. The insert diagrams in A–I are the frequency distributions of the Gini coefficients.

A total of 2449 street units (belonging to 152 county units) in 10 case cities were used to calculate the Gini coefficient of nine types of public facilities on the city scale. The Gini coefficients of educational and medical facilities were relatively similar, ranging from 0.23 to 0.88 and 0.23 to 0.89, respectively (Fig. 1). We found that among the various facilities, primary schools have relative equality (Gini = 0.38); middle schools, kindergartens, and pharmacies were between relative equality and inequality (0.4 < Gini < 0.5); and the remaining types of facilities have been in an inequality state (0.5 < Gini). Results indicated that the equality of facilities with small size and strong public attribute was higher than that of facilities with larger size or evident commercial attribute.

### Inequality of various public facilities in urban and rural areas

**Figure 2:**
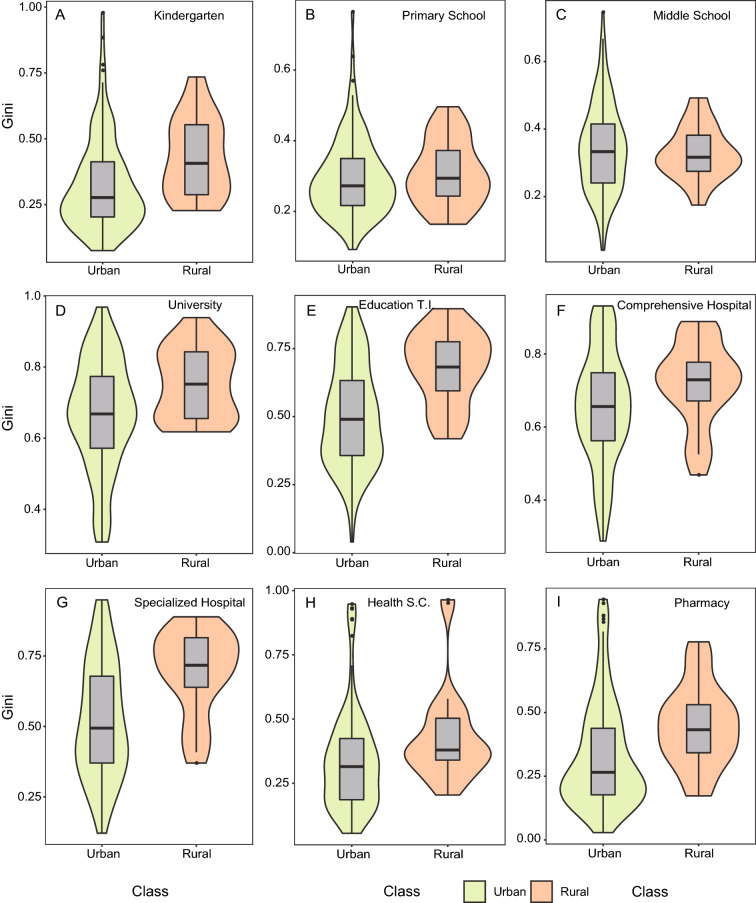
Comparison of the Gini coefficients for medical and educational facilities between urban and rural areas. Median across all points of analysis within a class is shown by a horizontal black line in A–I, with the 25th to 75th percentiles indicated by the box. The violin shapes filled with green and orange refer to the distribution of data in the urban and rural areas, respectively.

The Gini coefficient of urban areas represented by municipal districts was generally lower than that of rural areas represented by counties, except for middle schools (Fig. 2). Evidently, there remains a gap in the spatial equality of various educational and medical facilities between urban and rural areas. The median Gini coefficients for primary schools, middle schools, and health service centers were relatively equality (Gini < 0.4; Fig. 2B,C,H), and that for kindergartens and pharmacies were relatively equality in urban areas (Gini = 0.28, 0.26, respectively) but inequality in rural areas (Gini = 0.43, 0.43, respectively; Fig. 2A,I). However, for universities, education-training institutions, comprehensive hospitals, and specialized hospitals, urban and rural areas were in an inequality state (0.5 < Gini; Fig. 2D–G). Our result illustrated that facilities supported by the policies of nine-year compulsory education and basic medical security can guarantee equality in urban and rural areas.

**Figure 3:**
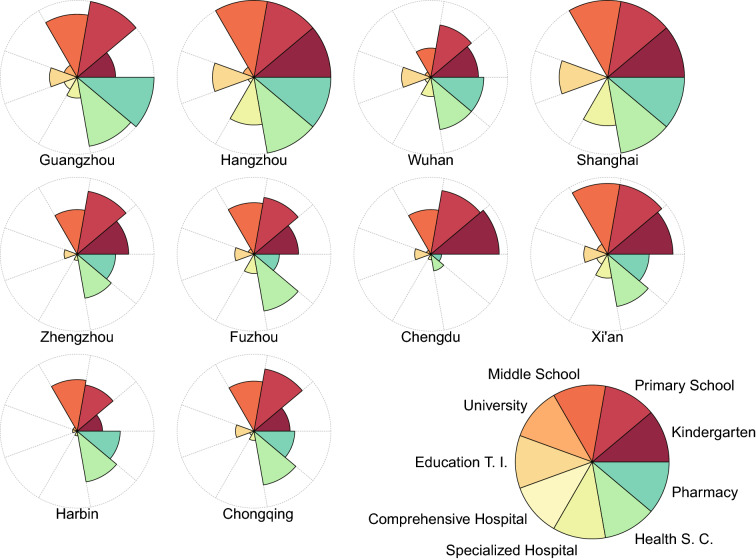
Proportion of regions with relative equality of the Gini coefficient for education and medical facilities in 10 cities. Petal length represents the quantification of the relatively equality proportion of the Gini coefficient for each type of facilities.

To understand the overall equality of various facilities in the spatial units (i.e., districts and counties) that constitute urban and rural areas, we analyzed the proportion of units that reach the equality threshold (Gini < 0.4) in each city (Fig. 3). Equality of various facilities was nearly consistent with the results of comparison between urban and rural areas. In 80% of the cities, over half of the districts and counties showed relative equality to facilities providing more basic education and medical services, such as kindergartens, primary schools, middle schools, health service centers, and pharmacies (Fig. 3). Significantly, there were still obvious differences in the proportion of the facility equity in specific cities. For instance, the aforementioned facilities were relatively equal in all counties in Shanghai and Hangzhou. However, for kindergartens in Harbin, middle schools in Wuhan, and pharmacies in Chengdu, the proportion of counties with relative equality was lower than that in other cities, which obviously has not reached 50%. Therefore, the results indicate that the allocation of public facilities may be closely related to the development characteristics of the city and policy support of the local government.

### Driving factors for spatial equality of public facilities

**Figure 4:**
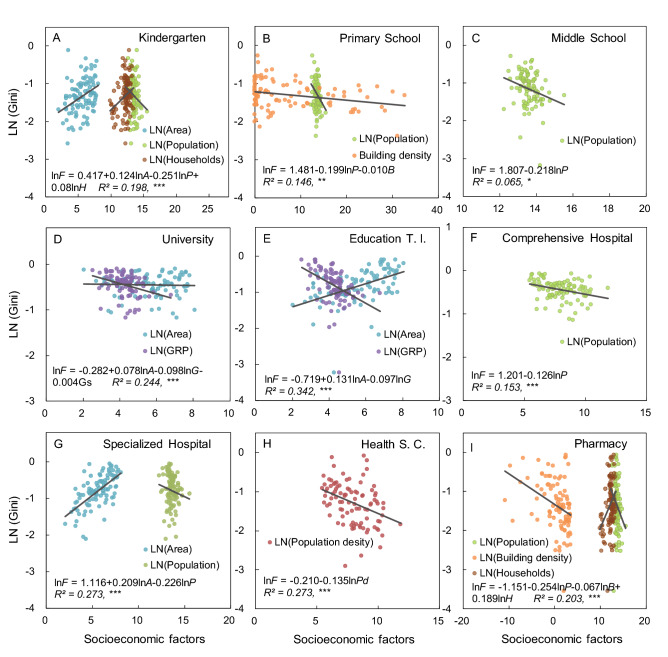
HPM and correlation regression between the Gini coefficient of various facilities and main socioeconomic factors. The color of the solid dot represents each socioeconomic factor. The solid lines indicate the trend of regression statistics. The formula in the figure represents the form of HPM, and the driving variables are represented in the form of abbreviations. The results of relevant regression coefficients are presented in the Appendix, Table S3. Significance level: *** *p* ≤ 0.001, ** *p* ≤ 0 0.01, * *p* ≤ 0.05.

Overall, stepwise regression analysis showed that the main driving factors of spatial equality of various facilities were different, involving regional area, population, gross regional product (GRP), households, and building density (Table 1). Population density was also significantly positively correlated with the equality of health service centers (Table 1). Given that we characterized equality based on matching degree between population and facilities, population variables were avoided to analyze the impact relationship. HPM for the Gini coefficient of various facilities and their main driving factors supported that the relationship exists in the form of logarithm and logarithmic linearity (Fig. 4).

**Table 1:** Stepwise linear regression selection results of the influence of socioeconomic factors on the Gini coefficients of various facilities.

Facilities	Factors	Estimate	*SE*	*t* value	*R*^2^ adj	*p* value
Kindergarten	Area	0.140	0.000	3.681	0.061	0.000
	Population	0.137	0.000	− 3.507	0.106	0.001
	Households	0.134	0.000	2.594	0.138	0.011
Primary School	Population	0.109	0.000	− 2.918	0.068	0.004
	Building density	0.105	0.001	− 2.864	0.135	0.005
Middle School	Population	0.127	0.000	− 2.430	0.050	0.017
University	Area	0.128	0.000	3.227	0.183	0.002
	GRP	0.125	0.000	− 2.916	0.223	0.004
Education T. I.	Area	0.155	0.000	6.481	0.286	< 0.001
	GRP	0.146	0.000	− 3.619	0.368	< 0.001
Comprehensive Hospital	Area	0.136	0.000	3.325	0.077	0.001
	Population	0.133	0.000	− 2.557	0.120	0.012
Specialized Hospital	Area	0.163	0.000	3.391	0.351	0.001
	Population	0.157	0.000	− 2.703	0.393	0.008
Health S. C.	Population density	0.165	0.000	− 2.229	0.041	0.028
Pharmacy	Building density	0.199	0.002	− 2.934	0.080	0.004
	Population	0.194	0.000	− 2.644	0.124	0.010
	Households	0.191	0.000	2.033	0.153	0.045

Gini coefficients of nine types of facilities were used as response variables, while eleven socioeconomic factors (refer to Stepwise regression analysis in the Methods) were used as explanatory variables. Only variables (*p* < 0.1) chosen in the final regression model when we used stepwise linear regression are shown.

## Discussion

Over the past three decades, China’s economic income inequality has been mainly reflected in regional differences and the gap between urban and rural areas^7,17,20^. For example, evident but insufficient empirical research has indicated that for education and medical resources, urban areas are often higher than rural areas, and the more developed coastal areas are also better than the less developed inland areas^1,17,31^. By using the Gini coefficient as an index, we determined the spatial equality of education and medical facilities in China’s 10 cities (Fig. 1; Appendix, Fig. S1). Our result preliminary illustrates that for most types of education and medical facilities, there are evident differences in equality between urban and rural areas (Fig. 2). In particular, how to solve the regional inequality caused by the urban–rural duality has become a critical problem hindering social development, but the premise is to accurately determine what type of facilities and determine the inequality^17,20^. We found that for educational and medical facilities, kindergartens and pharmacies demonstrated equality in urban areas but exhibited relative inequality in rural areas (Fig. 2A,I). Moreover, the equality of other types of facilities in the two regions was similar, either relative equality (e.g., primary schools, middle schools, and health service centers) or inequality (e.g., universities, education-training institutions, comprehensive hospitals, and specialized hospitals). However, there remained some differences in the degree of inequality (Fig. 2). To reduce the inequality of facilities between urban and rural areas, the government could emphasize on the “supplementary principle” before the “compensation principle” in mitigating the unequal distribution of public facilities^22^ (i.e., to solve the inequality of irreplaceable facilities, such as kindergartens, and consider facilities that can be temporarily replaced by other types of facilities, such as pharmacies). Furthermore, for large-size facilities that are unique to urban areas (e.g., universities, comprehensive hospitals, and specialized hospitals), many rural people can only enjoy services by paying travel costs or moving to urban areas^2,22^. Therefore, enhancing accessibility from rural areas to urban municipalities (such as convenient transportation facilities and lower welfare restrictions) is essential to reduce the equal difference in access to public facilities between urban and rural areas^17,51^. An irrelevant but interesting phenomenon is that the land surface temperature will increase with the increase of urban intensity in the urban–rural gradient^52^ (i.e., heat island effect), which indicates that residents in urban areas have to adapt to the adverse factors of corresponding environmental change while enjoying the advantages of public services.

Inequality among cities or regions with larger spatial scope has constantly existed^7,21^, which has also been confirmed by comprehensive comparative analysis of the Gini coefficients of various facilities in the representative cities with different urbanization rates of China’s four major economic zones in our study (Appendix, Fig. S2; Appendix, Table S1). The results showed that the equality of public facilities performs better in the eastern economic zone than in other regions. In recent years, the Chinese government has vigorously promoted the Western development strategy through the top–down path, and sufficiently focused on the construction of public facilities in underdeveloped regions^15,19,31^. This undertaking may explain that the facilities supported by the nine-year compulsory education policy and those serving the grassroots level, primary schools, middle schools, health service centers, and pharmacies can reach a relatively equal level in all regions (Appendix, Fig. S2). However, we admit that there are still some types of facilities that are significantly lower than the average in regions, such as kindergartens in the northeast, middle schools in the central region, and health service centers and pharmacies in the western region (0.4 < Gini < 0.5). In general, regions with better educational and medical resources (e.g., eastern region) can often attract more people, particularly wealthy people, to live and work in the region^1,42^. Conversely, areas lacking public facilities or with a high degree of inequality (e.g., western and northeast regions), population loss, or agglomeration of poor people often occur^43^, which leads to a non-virtuous cycle and hindering the further development of cities in the region. Therefore, only by improving the “short board” facilities in each region as much as possible can lay a basic guarantee for the improvement of the well-being of vulnerable areas and better realize the coordinated development of public facilities.

In general, spatial pattern of urban facilities depends on top–down planning and bottom–up spatial competition and also closely related to the functional attributes, quantity, and size of facilities^8,53^. Our results support the equality of educational and medical facilities with similar functions but small-size is significantly higher than that of large-size facilities (Fig. 1; Appendix, Fig. S3). Evidently, there is a considerable demand for the number of small-size facilities (i.e., primary schools, middle schools, and kindergartens; Appendix, Fig. S4), so their distribution is also more widely and evenly extensive^26,36^. Furthermore, the equality of facilities with evident public nature is generally higher than that of commercial facilities. For example, the size of education-training institution is relatively small, but their equality is poor compared with other educational facilities, second only to universities (Fig. 1A–E; Appendix, Fig. S1). In addition, primary and middle schools belong to nine-year compulsory education in China^15^. Therefore, as long as school-age children, their school needs will be guaranteed, which explains that their equality is higher than other types of educational facilities (Fig. 1B,C). Similarly, the degree of equality in medical facilities from high to low is health service centers, pharmacies, specialized hospitals, and comprehensive hospitals (Fig. 1F–I). Among them, the Gini coefficients of the first two types of facilities with smaller scale are about to reach relative equality, which is also due to the wide distribution in urban and rural areas and the government’s attention to basic medical facilities^40,47^. For facilities with large-size, such as universities, comprehensive hospitals, and specialized hospitals, their equality is relatively low (Fig. 1D,F,G; Appendix, Fig. S1), which is often caused by their clustered existence mode under the condition of limited urban land resources, resulting in strong spatial heterogeneity^45^. Although education-training institutions and pharmacies are small and have certain commercial attributes, the equality of pharmacies is significantly higher than that of education-training institutions (Fig. 1E,I). Note that the facilities dominated by bottom–up market competition (i.e., education-training institution), even if the number is high, its spatial distribution often has a certain degree of agglomeration^26,37^, so the equality will be low. Spatial inequality of benefit-driven facility distribution is reflected in the types of facilities and also in differences between urban and rural areas, as well as cities in different economic regions.

Equality of facilities assessed based on the Gini coefficient is closely related to the matching degree of population and is also affected by the attribute of urban social development^9,13^. We quantified HPM between various types of public facilities and socioeconomic factors that mainly affect their equality on the county scales (including districts), which is mainly shown in the form of logarithm and logarithmic linearity (Table 1; Fig. 4). This study is relatively more systematic and comprehensive than previous studies on the pattern and equality of certain types of facilities in cities^13,40^. We found a significant negative correlation between the regional area and spatial equality of various facilities (Table 1). In this study, regional area refers to the sub-urban scale (municipal districts and counties), and the rural area where the county is located is large but its population and corresponding facility demand are often small (Appendix, Table S1). Moreover, there is strong heterogeneity in the equality of facility distribution. Population and GRP, as important socioeconomic factors, were significantly positively correlated with facility equality (Table 1). In particular, population is a direct variable for calculating the Gini coefficient, and its impact on facility equality is self-evident^7,47^. Therefore, a reasonable idea is to avoid analyzing variables related to population, if not considering the need to integrate all impact factor analysis. Meanwhile, we found that the households had a significant correlation with the equality of kindergartens and pharmacies, which also reflected that the distribution of public facilities is closely related to the actual demands of residents. Previous studies have confirmed the important driving effect of GRP on the spatial pattern of urban facilities^26,36^ (e.g., urban–rural gradient). We further found that GRP has significant correlation with education-training institutions and universities on the county scales (Fig. 4). However, apart from the per capita GRP and urbanization rates, we did not find any other significant impact relationship between the equality of public facilities and socioeconomic factors on the city scales (Appendix, Figs. S5, S6). This also indicated that compared with the city scale, the smaller spatial scale (such as the county scale) can more clearly reflect the impact of driving factors on facility distribution^54,55^. Furthermore, building density had a significant logarithmic relationship with the spatial equality of pharmacies, although it did not show a certain correlation with that of other education and medical facilities (Table 1; Fig. 4). Similar research results indicated that building density may significantly affect the distribution of facilities on a more precise spatial scale, such as street scale^15,54^. Therefore, the impact of socioeconomic factors on distribution and equality of facility is different on different spatial scales^7,25^. Accordingly, exploring the driving mechanism of facility equality on multiple spatial scales (e.g., regions, cities, counties, and communities) may help to seek feasible suggestions conducive to urban development.

In our study, the gap in the equality of public facilities between case cities indicated that the coordinated development of education and medical facilities faces challenges under the differences of economic level and policy system (Figs. 1, 3), while case cities with good equality can also provide reference for sustainable development^4,43^. For example, the distribution of primary schools, middle schools, kindergartens, health service centers, and pharmacies in Shanghai and Hangzhou is nearly absolutely equal (Fig. 3). The reason is that, as an international metropolis^42^, the units of 17 districts and counties in Shanghai are in a relatively synchronous development state, except for the county of Chongming Island. Similarly, Hangzhou is the core city of the Yangtze River Delta urban agglomeration, and its Zhejiang province is the pilot area of the national “common prosperity” strategy^25^. However, for Chengdu, which is located in the Western Economic Zone, although the proportion of districts and counties with relatively equality basic education facilities is above 50%, the corresponding basic medical facilities (i.e., health service centers and pharmacies) have the lowest equality in the city (Fig. 3). In addition, our study found that even for cities with geographical and socioeconomic similarities, there was still an unequal relationship in the distribution of facilities and residents’ activities^23,24^. For instance, although the basic education facilities (i.e., kindergartens, primary schools, and middle schools) were relatively equal overall, some specific cities were still at a low level, such as kindergartens in Harbin and middle schools in Wuhan (Fig. 3). Therefore, for the education and medical facilities, the local government should focus considerably on the types of facilities that are highly unequal but can still be improved^15,40^, thereby gradually improving the equality of local residents’ access to facility resources. For example, the expanded university campuses can be gradually distributed to the suburbs or small cities around the metropolis, so as to relatively alleviate the equality difference of higher education between regions in the city^15,22^. However, how to balance regions with relatively backward economic development and a huge gap in the ability to obtain scarce services with cities in the southeast coastal region remain a challenge for regional coordinated development.

At present, some urban transformation is still in progress, which is mainly reflected in the aggregation of rural young labor force to urban areas^44,51^, and accompanied by an increase in demand for education and health services^47^. In recent years, the Chinese government has appropriately reduced the restrictions on children of urban migrants to enjoy urban benefits in urban areas^17^, and liberalized the two-child and three-child policies to adjust the population age structure^19^, which will have a certain impact on the spatial equality of education and medical infrastructure in urban and rural areas^21^. On the one hand, although the distribution of basic education facilities in urban and rural areas, such as primary and middle schools, has reached a relatively equal level (Fig. 2; Appendix, Fig. S2), more “rural children” in urban areas may still face the dilemma of being unable to go to school in the future. On the other hand, the increase in the number of newborns is bound to increase the demand for kindergartens and corresponding medical facilities, so the existing supporting facility planning formulated by local governments may be inapplicable to the development needs, particularly in some cities with backward facility equality on a county scale, such as Zhengzhou, Chengdu, and Harbin (Fig. 3). More importantly, the population agglomeration in urban areas leads to a shortage in education and medical facilities, while land resources for urban development are limited^15,22^. Most villages have basic public facilities (e.g., kindergartens, primary schools, middle schools, and health service centers), but the demand population is gradually losing (especially school-age children) and the corresponding talent resources of educational and medical are decreasing^7,17,31^. To alleviate the inequality of social resources and realize the equalization of public service facilities, the matching of supply and demand facility resources^22^, the corresponding national development strategies^56^, as well as the difficulties faced by local urban development should be comprehensively considered^13,17^. Social sustainability^2,7^ (e.g., facilities services, income, and welfare) and environmental sustainability^11,52^ (e.g., air quality, climate change, and green space) are crucial in building livable and equitable cities that can be maintained in the long term^13^. Improving social equality means that all residents, particularly for vulnerable groups and low socioeconomic groups, have access to social and natural services^43^. Therefore, an improved understanding of the inequality of various resources related to human socioeconomic activities is conducive to improving the well-being of residents in developing countries and even developed countries, and further achieving sustainable urban development.

However, there are still some limitations that should be further improved in this study. First, this study defined the main types of educational and medical facilities according to statistical yearbooks and industrial classification standards, but a few facilities were not included^36,55^ (e.g., private clinics, pet hospitals, higher vocational colleges, and driving schools). For further research, a more detailed and systematic evaluation may be carried out according to the functional type of the facility. Second, we analyzed the equality of facility by taking the individual facilities as mass points (hospitals, universities, and other facilities took independent buildings as the representative of quantity), disregarding the differences in the scale and service radius between facilities^2,25^, which is mainly limited by data acquisition. For the future work, the parameters related to the facility attributes and accessibility can be considered, and the supply–demand balance of facility services can be measured based on the existing equity framework^22^ (such as the integrated spatial equity evaluation framework), which is conducive to a more comprehensive and reasonable measurement of the equality of facilities. Third, owing to the limited access to urban electronic map data, 10 case cities are currently used to represent various economic regions in China, which is not considerably comprehensive. With the same limitation, we have not obtained the latest year’s data of facilities (such as 2020). To more systematically reflect the equality of various facilities in China, further research can consider analyzing from the perspective of spatiotemporal dynamics, and appropriately expanding the number of case cities. In addition, there is no unified and clear definition of rural areas in previous studies^30,32,33^. Hence, we regarded areas outside the boundary of urban areas as rural areas, which may include areas unrelated to human economic activities, and may lead to a certain deviation in analyzing the correlation between area and facility equity. Lastly, our research tends to assess the equality of public facilities from the urban and regional levels, but cannot be extrapolated to the individual level^2,44^. Therefore, the parameters that can represent the attributes, demands and socioeconomic activities of residents were not considered, such as population characteristics, education level, land or housing prices, and residents’ personal preferences. Further research can consider acquiring and integrating these parameters to explore the driving mechanism of facility equality more deeply, contribute to urban planning and management, and promote the improvement of human well-being.

## Conclusion

This study mainly analyzes the inequality of various education and medical facilities between urban and rural areas in cities with different development levels. Compared with previous relevant studies focusing on the spatial equality of a single or few types of educational or medical facilities within a city, the comparative analysis of various types of public facilities on multiple spatial scales can promote the awareness of social inequality and contribute to the systematic and scientific planning of public facilities. The results indicated that compared with facilities with large-size or evident commercial attributes (i.e., universities, education-training institutions, comprehensive hospitals, specialized hospitals, and pharmacies), facilities with public attributes (i.e., kindergartens, primary schools, middle schools, and health service centers) showed relatively good equality in spatial distribution, whether in urban or rural areas. A seemingly common fact is that there remain significant differences in facility inequality between urban and rural areas, especially the facilities that are relatively equal in urban areas but inequality in rural areas (i.e., such as kindergartens and pharmacies). The identification results of the types and regions of facilities with unequal distribution showed that the inequality of public facilities performs better in the eastern economic zone than in other regions. In addition, we found that in most cases, more than half of the county level units have relatively equal basic public facilities, except for kindergartens in Harbin, middle schools in Wuhan, and pharmacies in Chengdu. These findings will help mankind deeply understand the equality of public facilities in urban and rural areas and identify the types and regions of facilities with unequal distribution, which will provide scientific support for the government to reasonably adjust and manage public facilities and further promote regional coordinated development. Last but not least, the inequality of public facilities at the county level is mainly affected by population, gross regional product, and building density. Evidently, in order to improve the well-being of residents, the urban development requires an in-depth understanding of the driving mechanisms of human socioeconomic activities on the equality of public facilities, and inevitably learns from the experience of different cities, regions, and countries.

## Methods

### Study regions

**Figure 5:**
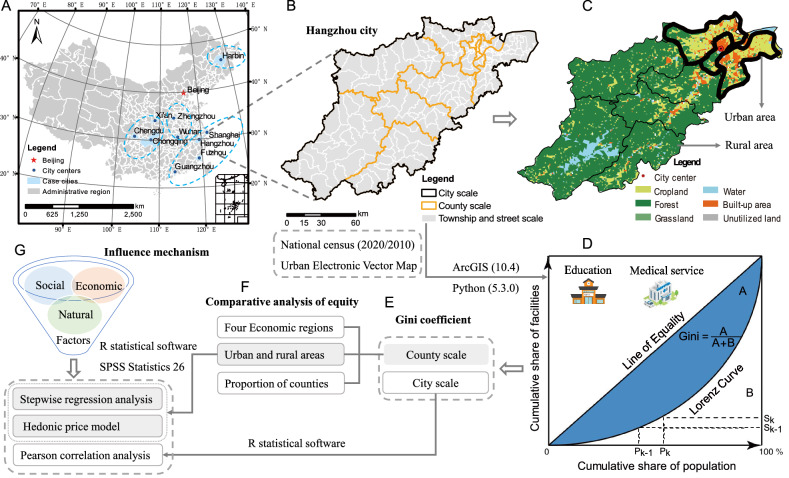
Study regions and framework of the research methods. (**A**) Geographical distribution of the 10 case cities in China. (**B**) Administrative divisions at all levels of a city are illustrated by taking Hangzhou as an example. (**C**) Land use classification of Hangzhou is used to clearly distinguish urban and rural areas. (**D**) Graphical definition of urban facility equality. (**E**–**G**) Calculation of facility equality coefficient and analysis of impact mechanism and the gray shadows indicate emphasis content.

This study was conducted in 10 major provincial capitals or municipalities in China: Shanghai, Chongqing, Guangzhou, Hangzhou, Wuhan, Fuzhou, Zhengzhou, Chengdu, Xi’an, and Harbin (Fig. 5A). The selection criteria of the representative cities are as follows: (1) including at least one international metropolis or municipality directly under the central government; (2) covering the four major economic zones of Eastern, Central, Western, and Northeast China; (3) representing different economic levels, urbanization rates, and population sizes; and (4) containing at least 10 administrative municipal districts or counties. We used seven common socioeconomic and environmental factors to represent the basic urban attributes: urban population (person), population density (person km^−2^), gross regional product (GRP, yuan year^−1^), per capita GRP (yuan per^−1^ year^−1^), built-up area (m^2^), urbanization rates (%), and green space coverage (%). Data were retrieved from the *China City Statistical Yearbook*^26^ in 2017 (http://tongji.cnki.net/), referred to Appendix, Table S1 for details. The determination of the year mainly depends on the data of urban facility obtained (see the part of facility data acquisition for detailed explanation), and the years of other types of data need to be consistent with that year (2016) as much as possible. Spatial scale of the city was divided into three levels (Fig. 5B): city, county (i.e., municipal districts and counties), and street (i.e., townships and streets) scales (refer to Appendix, Table S2 for details of the case cities). We took the administrative boundary of the municipal districts as boundary of the urban areas, and listed the areas outside the boundary as the rural areas of the city^9,11^ (Fig. 5C). The criteria used to discern urban and rural areas mainly consider the clarity of administrative boundaries and the feasibility of policy recommendations based on research results. Boundaries of cities and counties (or districts) were based on the administrative division of China, and the 2015 data were from the Database of Global Administrative Areas^57^ (https://gadm.org/). The 2015 boundary data of the street scale were from the Database National Earth System Science Data Center (http://www.geodata.cn/).

### Description and acquisition of educational and medical facilities

Urban public facilities in this study are defined as buildings or equipment provided by the government or other social organizations and used or enjoyed by the public, including educational, medical and health, cultural, and entertainment facilities^2,22^. We took educational and medical facilities as the representative of urban public facilities, which is attributed to the fundamental role of the two types of facilities in human survival and development^1,17,31^. On the basis of the classification of urban facilities in the *China City Statistical Yearbook*^26^ in 2017, educational facilities are divided into four categories: kindergartens, primary schools, middle schools (including junior and senior high schools), and universities. Medical facilities are divided into three categories: comprehensive hospitals, specialized hospitals, and health service centers. Meanwhile, facilities with educational and medical functions also drive the development of some commercial facilities driven by the market^36^. Therefore, profit education-training institutions and retail pharmacies may also be included in educational and medical facilities, respectively. Accordingly, we defined and described the terms of nine types of educational and medical facilities used for the equality analysis of urban public facilities (Table 2).

**Table 2:** Description of educational and medical facilities.

Urban facilities	Descriptions
**Education facilities**	Providing systematic educational activities to the educated in a planned and organized manner
Kindergartens	Providing preschool education, and school-age children are generally 3 to 6 years old
Primary Schools^a^	Providing primary formal education, and school-age children are generally 6 to 12 years old
Middle Schools^a^	Providing secondary formal education, including junior middle and senior high schools, and school-age students are generally 13 to 18 years old
Universities	Providing higher education, including comprehensive universities, specialized universities, and colleges, and school-age students are generally 18 to 22 years old
Education-training Institutions^b^	Institutions or websites for academic education, including skills and business trainings
**Medical facilities**	Providing diagnosis, medicine, medical equipment, ward accommodation, and other services for people in need of health treatment
Comprehensive Hospitals	Dealing with various diseases and injuries, often including emergency, outpatient, and inpatient departments
Specialized Hospitals	Dealing with specific diseases or injuries, including pediatric, gynecological, and dermatological hospitals
Health Service Centers	Providing convenient and basic medical services, such as prevention, health care, medical services, and health education for communities, families, and residents
Pharmacies^b^	Providing retail drugs to facilitate people’s purchase of drugs

^a^Primary and junior middle schools are 9-year compulsory education, but senior middle schools are non-compulsory education.

^b^Education-training institutions and pharmacies provide education and medical services, respectively, and most of them belong to profit-making facilities.

This study has high requirements for the accuracy of the data of urban facilities (including names and locations). The data of 2016 Urban Electronic Vector Map has a high resolution (http://www.dsac.cn/), and the accuracy has been proved through the investigation and empirical verification in relevant studies^34,53^. Given the differences in the scale of various educational and medical facilities, for facilities containing more than one building (e.g., universities and comprehensive hospitals), we took each building with specific functions as the statistical unit of such facilities^53^. First, we used the “spatial join” tool in ArcGIS (10.4) to associate with administrative map on the city, county, and street scales (Fig. 5). Second, we used the street scale boundary map as basis in utilizing the ArcGIS tool to extract facility information. Third, we divided all educational and medical facilities into nine categories according to the name and primary classification code in the tables of facility attributes, combined with visual interpretation. Lastly, we obtained information of various educational and medical facilities on the street scale in all case cities.

### Quantify and mapping the equality of facilities

#### Drawing of Lorenz curve

Before the calculation of the Gini coefficient, we need to draw the Lorenz curves of educational and medical facilities based on their cumulative number and population on the street scale (Fig. 5D). The number of facilities was obtained through the method described in the previous step. Population data on street scale was derived from the Seventh National Census (2020) published by various urban statistical bureaus (e.g., Hangzhou Bureau of Statistics; http://tjj.hangzhou.gov.cn). Given that the census data of 2020 for most cities only disclose at the district and county levels, we obtained the 2016 data for cities lacking township and street scale data by converting the annual growth rate, combined with the data of the Sixth National Census (2010) released by the National Bureau of Statistics (http://www.stats.gov.cn). After ranking the per capita resources of street units from low to high according to the population and number of various facilities of each street unit, we took the accumulated population as the abscissa and the corresponding cumulative amount of various educational and medical facilities as the ordinate to draw the Lorenz curves of facility resources in each city. To analyze the equality of facilities between urban and rural areas, we treated all districts and counties of each city in the same manner.

#### Calculation of the Gini coefficient

Given that the Lorentz curve is not a smooth curve, there are numerous formulas to choose when actually calculating the Gini coefficient. The Gini coefficients of per capita facility resources were calculated as follows^9^:

1 $$Gini=A/\left(A+B\right),$$

where *A* is the area between the Lorenz curve and line of absolute equality, and *B* is the area under the Lorenz curve. Thus, their sum constitutes the area of a right triangle under the line of absolute equality (Fig. 5D). The Gini coefficient ranges from 0 (absolute equality) to 1 (absolute inequality). The area under the Lorenz curve (marked *B* in the diagram) was calculated as follows:

2 $$B=\frac{\sum_{k=1}^{n}({P}_{k}-{P}_{k-1})({S}_{k}+{S}_{k-1})}{2},$$

where *n* represents the number of street units, which is sorted and numbered according to the per capita facility resources from low to high; the value of *k* ranges from 0 to *n*; ${P}_{k}$ is the cumulative proportion of the population, ${P}_{0}$ = 0, ${P}_{n}$ = 1; and ${S}_{k}$ is the cumulative proportion of the number of various facilities acquired by the people corresponding street units, ${S}_{0}$ = 0, ${S}_{n}$ = 1. Therefore, the area that lies between the line of absolute equality and the coordinate axis is 0.5, and *A* was calculated as follows:

3 $$A=0.5-B.$$

Combining the preceding three formulas, the Gini coefficients can be broken down as follows:

4 $$Gini=1-\sum_{k=1}^{n}\left({P}_{k}-{P}_{k-1}\right)\left({S}_{k}+{S}_{k-1}\right),$$

where the meanings of the parameters are the same as those in Eq. (2). The Gini coefficient is divided into five levels: absolute equality (0 ≤ Gini < 0.2), comparative equality (0.2 ≤ Gini < 0.3), relative equality (0.3 ≤ Gini < 0.4), inequality (0.4 ≤ Gini < 0.5), and very inequality (0.5 ≤ Gini ≤ 1). Internationally, a coefficient of 0.4 is often taken as the warning line of distribution gap. Each type of facilities can be displayed by a Lorentz curve on the city and county scales, and each curve corresponds to a Gini coefficient value. For educational and medical facilities, we calculated the frequency distribution histograms of the Gini efficiency in case cities to assist in judging the equality of various facilities.

#### Comparative analysis of the Gini coefficient on multiple spatial scales

We conducted a comparative analysis of facility equality from three scenarios (Fig. 5E,F). First, we analyzed the difference of the average Gini coefficient between urban and rural areas. We used the Gini coefficient calculated by street scale as basis in analyzing the differences between urban and rural areas by dividing municipal districts and counties^25^ (Fig. 5C). Second, we analyzed the proportion of municipal districts and counties with relative equality of various education and medical facilities in the case city (i.e., ratio of the number of districts and counties with Gini coefficients below 0.4 calculated based on the total number of districts and counties in the city). This analysis is used to identify the types of facilities with inequality distribution at the district and county levels in each city. Third, we explored the equality disparity of cities in different economic and geographical regions of China (i.e., four major regions: eastern, western, central, and northeast regions) according to the regional division of the National Bureau of Statistics of China. In this scenario, the calculation of the Gini coefficients based on city scale (i.e., each type of facility in each city has one Gini coefficient value).

### Analysis of the driving mechanism of socioeconomic factors on facility equality

#### Stepwise regression analysis

To accurately identify the main drivers affecting the spatial equality of various education and medical facilities, we carried out a stepwise regression analysis using the Gini coefficients of facilities as response variables and driving factors as explanatory variables on the district and county scales (Fig. 5G). We used eleven socioeconomic factors to represent attributes of districts and counties: urban population (person), population density (person km^−2^), households (household), sex ratio of population (%), gross regional product (GRP, yuan year^−1^), per capita GRP (yuan per^−1^ year^−1^), per capita disposable income (yuan per^−1^ year^−1^), region area (m^2^), building density (%), building height (m), and green space coverage (%). Among them, data of building (2017) and vegetation coverage (i.e., normalized difference vegetation index; 2015) were derived from the Resource and Environment Science and Data Center (http://www.resdc.cn/). Other types of attribute data were derived from the *China City Statistical Yearbook*^26^ in 2017 (http://tongji.cnki.net/).

In addition, we further revealed the driving mechanism of socioeconomic and natural factors on facility equality through the Pearson correlation analysis between the Gini coefficient of various facilities and urban attributes on the city scale (see Study regions in the Methods for the seven urban attributes). Note that although the driving factors include parameters related to population, avoiding a certain correlation is difficult because the Gini coefficient is also measured by population. The stepwise regression and Pearson correlation analyses were conducted using R statistical software (version 3.1.2).

#### Construction of HPM

We used the main driving factors determined by stepwise regression analysis as bases in using HPM to reveal the comprehensive impact of multiple socioeconomic factors on the equality of various education and medical facilities. HPM often adopts three equation forms: linear, logarithmic, and logarithmic linear^49^. According to previous studies, the single factor has a linear or logarithmic relationship with the density and diversity of facility distribution^26,53^. Hence, we carried out an HPM with the logarithmic or logarithmic linear form, in which the independent variable is a major driving factor and the dependent variable is the Gini coefficients of facilities. Accordingly, we assumed, at most, three major factors for each type of facility. In HPMs, continuous variables were utilized in the logarithmic form. HPM in this study is defined as follows:

5 $$InQ={\alpha }_{0}+\sum {\alpha }_{i}In{Z}_{i}+\sum {\alpha }_{j}{Z}_{j}+\sigma ,$$

where *Q* is the value of the Gini coefficients, ${Z}_{i}$ is the variable in logarithmic form of the driving factors and ${Z}_{j}$ is the variable in linear form of the driving factors, ${\alpha }_{0}$ is an intercept term, ${\alpha }_{i}$ and ${\alpha }_{j}$ are the coefficients of the corresponding variables, and $\sigma$ is an error term. When HPM is expressed as a logarithmic model, the value of ${\alpha }_{j}$ is zero.

To ensure the applicability of HPM, we needed to test the simulation results of the model. First, the significance probability of the *F* statistical value of analysis of variance should be under 0.05, and the characterization equation is generally significant. Second, the higher the value of the adjusted *R*^2^, the better the fitting degree and interpretation ability of the model. Third, the significance level of the t-test of the regression coefficient of the model should be below 0.05. For the collinearity test, the *VIF* values of all variables are under 10. Hence, the degree of multicollinearity between independent variables is considered not large. Lastly, we built HPM that can reflect the impact of various main driving factors on the equality coefficients of nine types of education and medical facilities. The analyses are conducted using the software IBM SPSS Statistics 26 (https://www.ibm.com/products/spss-statistics).

## Supplementary Information

Supplementary Information.

## Data Availability

The data that support the findings of this study are available from the corresponding author upon reasonable request.
