# Supplementary material for: Inequality of public facilities between urban and rural areas and its driving factors in ten cities of China
Source: Sci Rep. 2022 Aug 2;12:13244. doi: 10.1038/s41598-022-17569-2 (PMC9344805; doi:10.1038/s41598-022-17569-2)
Supplement: Supplementary file 1 — Supplementary Information. [file 41598_2022_17569_MOESM1_ESM.docx]

**Inequality of public facilities between urban and rural areas and its driving factors in ten cities of China**

**Supplementary information**

Ronghua Xu^1^, Wenze Yue ^1^*, Feiyang Wei^2^, Guofu Yang^3^, Yi Chen^4^, Kaixuan Pan^5^

^1^ *Department of Land Management, Zhejiang University, Hangzhou 310058, PR China*

^2^*School of Design and Environment, National University of Singapore, Singapore 117566, Singapore*

^3^*Artistic Design & Creation School, Zhejiang University City College, Hangzhou 310015, PR China*

^4^*College of Life Sciences, Zhejiang University, Hangzhou 310058, PR China*

^5^ *Institute of Environmental Science, Leiden University, Einsteinweg 2, 2333, CC Leiden,* *The Netherlands*

The corresponding author’s* email addresses: wzyue@zju.edu.cn

Tel & Fax: +86-571-5666 2160

Postal addresses: Department of Land Management, Zhejiang University

866 Yuhangtang Road, Hangzhou, 310058, Zhejiang Province, PR China

**This file contains:**

Table S1-S3

Figure S1-S6

**Supplementary Table S1.** Urban attributes for ten case cities in China

| City | Population (10^6^) | Density (person/km^2^) | GRP (10^9^ yuan) | Per capita GRP (10^3^ yuan) | City area (m^2^) | District area (m^2^) | Built-up area (m^2^) | Urbanization rate (%) | Green space coverage (%) |
| --- | --- | --- | --- | --- | --- | --- | --- | --- | --- |
| Chengdu | 12.2 | 1013.1 | 1080.1 | 74.3 | 12121 | 3240 | 616 | 70.6 | 39.8 |
| Chongqing | 33.7 | 409.0 | 1571.7 | 52.3 | 82374 | 34505 | 1329 | 62.6 | 40.3 |
| Fuzhou | 6.8 | 534.9 | 561.8 | 75.3 | 12675 | 1786 | 260 | 68.5 | 43.4 |
| Guangzhou | 13.5 | 1816.0 | 1810.0 | 136.2 | 7434 | 7434 | 1237 | 86.1 | 41.6 |
| Hangzhou | 7.2 | 436.3 | 1005.0 | 112.2 | 16596 | 4876 | 506 | 76.2 | 40.4 |
| Harbin | 9.7 | 181.0 | 575.1 | 59.0 | 53100 | 10198 | 428 | 64.0 | 31.6 |
| Shanghai | 24.2 | 2275.7 | 2512.3 | 103.8 | 6341 | 6341 | 1500 | 87.6 | 38.1 |
| Wuhan | 8.3 | 967.4 | 1090.6 | 104.1 | 8569 | 1738 | 455 | 79.8 | 42.5 |
| Xi′an | 8.8 | 875.0 | 580.1 | 66.9 | 10097 | 3874 | 501 | 73.4 | 42.6 |
| Zhengzhou | 8.0 | 1087.8 | 731.2 | 77.2 | 7446 | 1010 | 438 | 71.0 | 40.3 |

Note: The variables related to population and GRP are of the whole city. Data obtained from China City Statistical Yearbook in 2017 (<http://tongji.cnki.net/>).

**Supplementary Table S2.** Grouping and administrative division of ten case cities

| City | Economic regions | Districts or counties | Townships or streets |  |
| --- | --- | --- | --- | --- |
|  |  |  |  |  |
| Guangzhou | Eastern | 11 | 167 |  |
| Hangzhou | Eastern | 13 | 195 |  |
| Wuhan | Central | 13 | 198 |  |
| Shanghai | Eastern | 16 | 225 |  |
| Fuzhou | Eastern | 13 | 193 |  |
| Chengdu | Western | 19 | 313 |  |
| Harbin | Northeast | 18 | 316 |  |
| Zhengzhou | Central | 12 | 194 |  |
| Xi′an | Western | 13 | 174 |  |
| Chongqing | Western | 24 | 472 |  |

Note: Data was extracted from the administrative division boundary obtained from the National Earth System Science Data Center (http://www.geodata.cn/).

**Supplementary Table S3.** Regression results of HPM

| Variables | *USC* | *SE* | *SC* | *t value* | *Sig.* | *VIF* |
| --- | --- | --- | --- | --- | --- | --- |
| **Kindergarten** |  |  |  |  |  |  |
| Constant term | 0.417 | 1.310 |  | 0.319 | 0.751 |  |
| Area | 0.124 | 0.031 | 0.386 | 4.047 | 0.000 | 1.069 |
| Population | -0.251 | 0.086 | -0.274 | -2.914 | 0.004 | 1.033 |
| Households | 0.080 | 0.053 | 0.140 | 1.492 | 0.139 | 1.036 |
| **Primary School** |  |  |  |  |  |  |
| Constant term | 1.481 | 0.954 |  | 1.552 | 0.124 |  |
| Population | -0.199 | 0.070 | -0.273 | -2.830 | 0.006 | 1.003 |
| *Building density* | -0.010 | 0.004 | -0.252 | -2.613 | 0.010 | 1.003 |
| **Middle School** |  |  |  |  |  |  |
| Constant term | 1.807 | 1.164 |  | 1.552 | 0.124 |  |
| Population | -0.218 | 0.086 | -0.255 | -2.546 | 0.013 | 1.000 |
| **University** |  |  |  |  |  |  |
| Constant term | -0.282 | 0.202 |  | -1.393 | 0.167 |  |
| Area | 0.078 | 0.019 | 0.471 | 4.041 | 0.000 | 1.635 |
| GRP | -0.098 | 0.035 | -0.280 | -2.822 | 0.006 | 1.187 |
| **Education T. I.** |  |  |  |  |  |  |
| Constant term | -0.719 | 0.338 |  | -2.128 | 0.036 |  |
| Area | 0.131 | 0.028 | 0.419 | 4.708 | 0.000 | 1.110 |
| GRP | -0.197 | 0.059 | -0.297 | -3.328 | 0.001 | 1.110 |
| **Comprehensive Hospital** |  |  |  |  |  |  |
| Constant term | 1.201 | 0.595 |  | 2.020 | 0.046 |  |
| Population | -0.126 | 0.044 | -0.278 | -2.866 | 0.005 | 1.019 |
| **Specialized Hospital** |  |  |  |  |  |  |
| Constant term | 1.116 | 0.900 |  | 1.240 | 0.218 |  |
| Area | -0.226 | 0.067 | -0.259 | -3.368 | 0.001 | 1.033 |
| Population | 0.209 | 0.023 | 0.685 | 8.908 | 0.000 | 1.033 |
| **Health S. C.** |  |  |  |  |  |  |
| Constant term | -0.210 | 0.283 |  | -0.744 | 0.459 |  |
| Population density | -0.135 | 0.035 | -0.372 | -3.871 | 0.000 | 1.000 |
| **Pharmacy** |  |  |  |  |  |  |
| Constant term | -0.151 | 1.793 |  | -0.084 | 0.933 |  |
| Building density | -0.067 | 0.020 | -0.317 | -3.384 | 0.001 | 1.033 |
| Population | -0.254 | 0.118 | -0.201 | -2.157 | 0.034 | 1.028 |
| Households | 0.189 | 0.072 | 0.241 | 2.611 | 0.011 | 1.008 |

Note: All variables are in logarithmic form, except that those in italics in the leftmost column are linear. *USC* means unstandardized coefficient, *SE* is standard error, and *SC* is standardized coefficient. For the regression coefficient, *p* < 0.05 and *VIF* < 10 in all models.

**Supplementary Figure S1**. The mean value of Gini coefficient of public facilities in all case cities. The Gini coefficients reflect the equality of nine types of public facilities in the entire city.


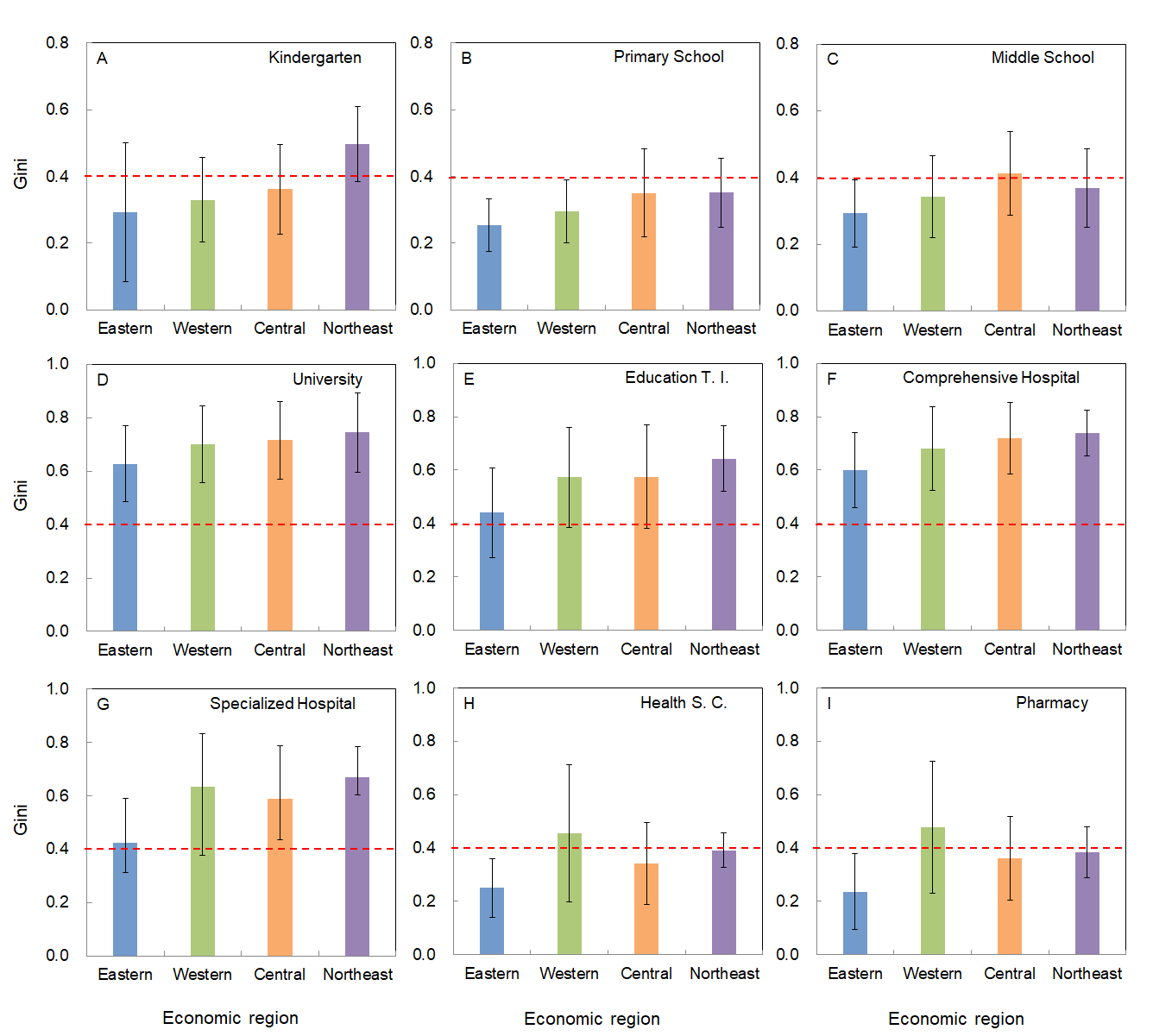


**Supplementary Figure S2.** Comparison of the Gini coefficients for medical and educational facilities among China’s four economic regions. Data of statistical units of sub-urban scale (districts and counties) in each region is provided in *Appendix*, Table. S2.


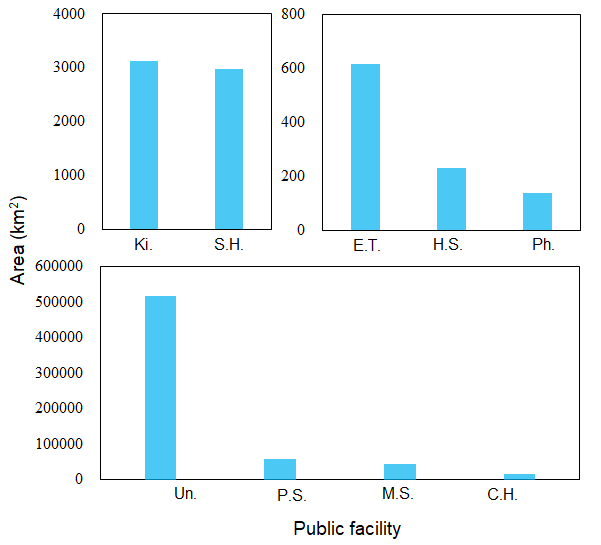


**Supplementary Figure S3.** Base areas of the representative public facilities. The data of the average area of nine types of public facilities is obtained from the field investigation in Hangzhou City (n=35). The abbreviations letters of the abscissa axis represent various facilities, Ki. (Kindergartens), S.H. (Specialized hospitals), E.T.I. (Education-training institutions), H.S.C. (Health service centers), Ph. (Pharmacies), Un. (Universities), P.S. (Primary schools). M.S. (Middle schools), and C.H. (Comprehensive hospitals), respectively.

**Supplementary Figure S4**. The quantity and proportion of public facilities in case cities. The unit of proportion is percentage (%).


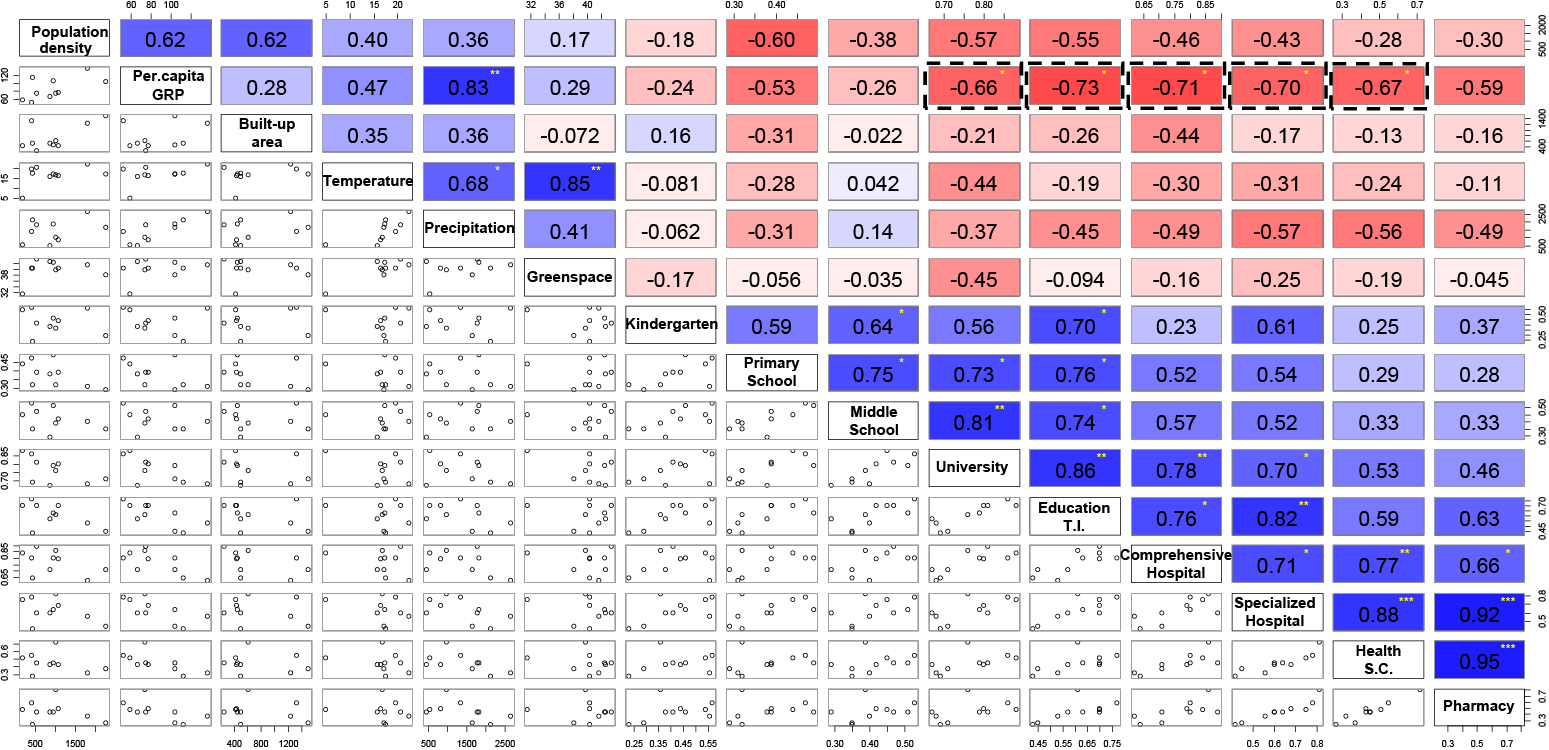


**Supplementary Figure S5.** Pearson correlations among urban attributes and Gini coefficients of various facility. The Gini coefficients reflect the equality of public facilities in the entire city. Blue and red colors indicate positive and negative correlations, respectively. The dotted black box indicates the significant correlation value of the emphasis. Significance level: *** *p* ≤ 0.001, ** *p* ≤ 0.01, * *p* ≤ 0.05


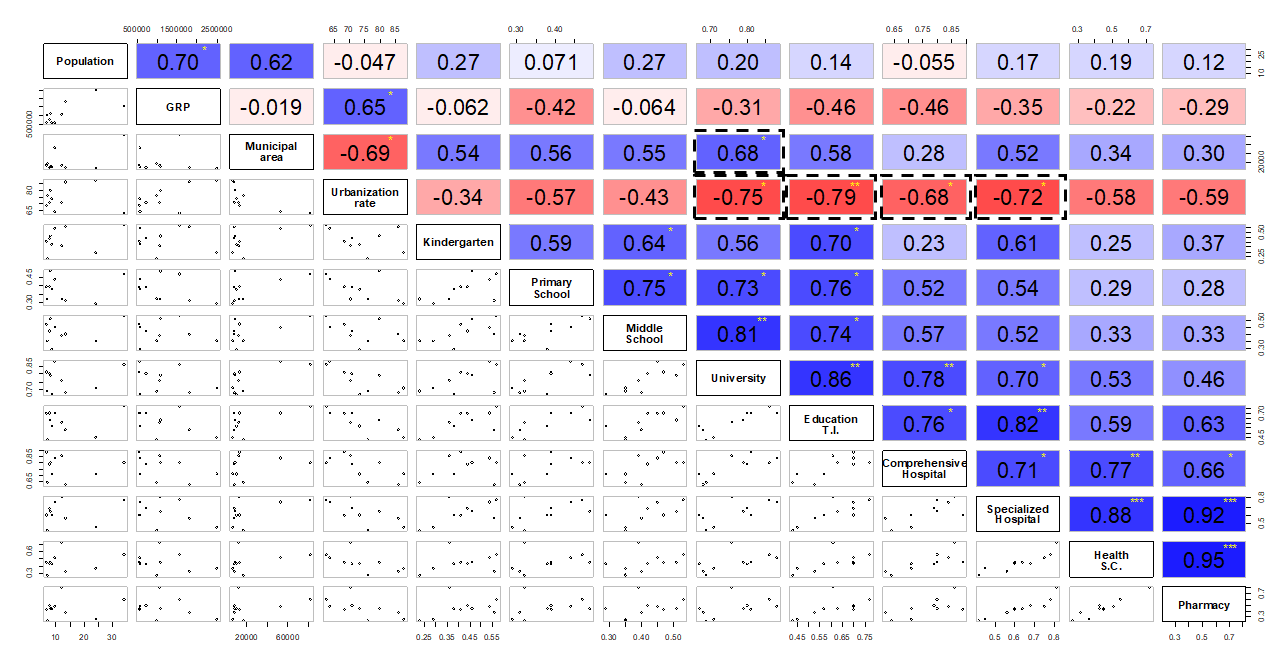


**Supplementary Figure S6.** Pearson correlations among urban attributes and Gini coefficients of various facility. Blue and red colors indicate positive and negative correlations, respectively. Significance level: *** *p* ≤ 0.001, ** *p* ≤ 0.01, * *p* ≤ 0.05
